# Supplementary material for: 5-Aryl-1-Arylideneamino-1H-Imidazole-2(3H)-Thiones: Synthesis and In Vitro Anticancer Evaluation
Source: Molecules. 2021 Mar 18;26(6):1706. doi: 10.3390/molecules26061706 (PMC8003321; doi:10.3390/molecules26061706)
Supplement: Supplementary file 1 [file molecules-26-01706-s001.zip › molecules-1123643-supplementary.pdf]

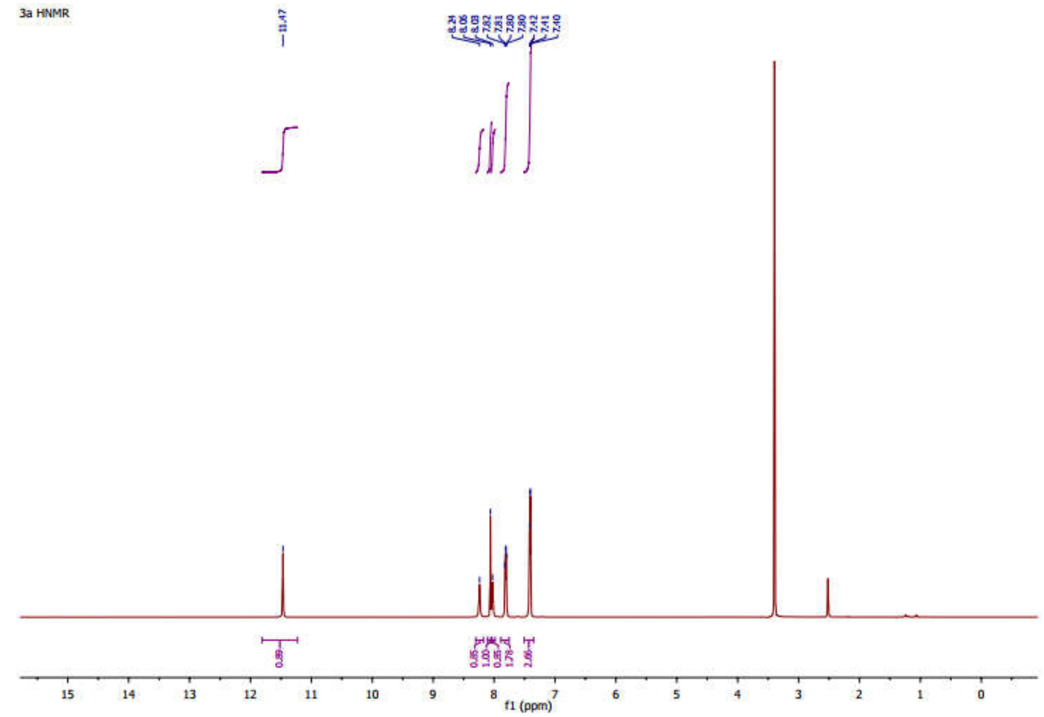

Fig. 1: ^{1}H-NMR spectrum of compound 3a

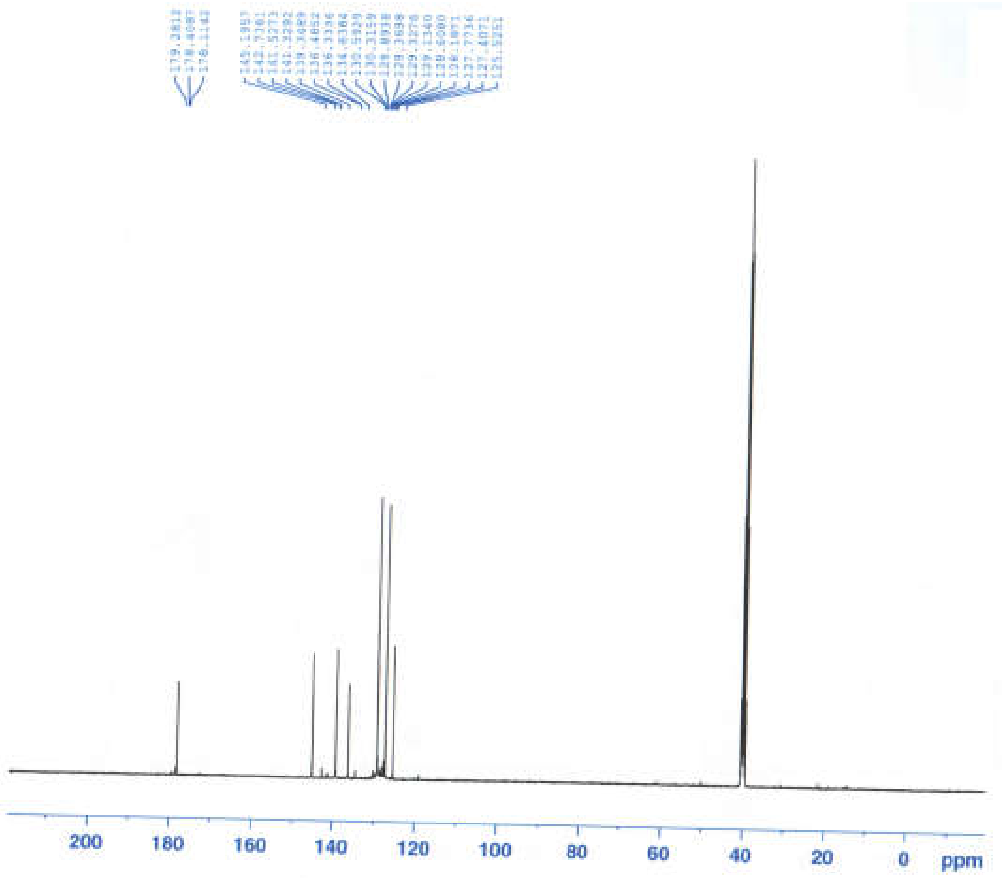

Fig. 2: ^{13}C-NMR spectrum of compound 3a

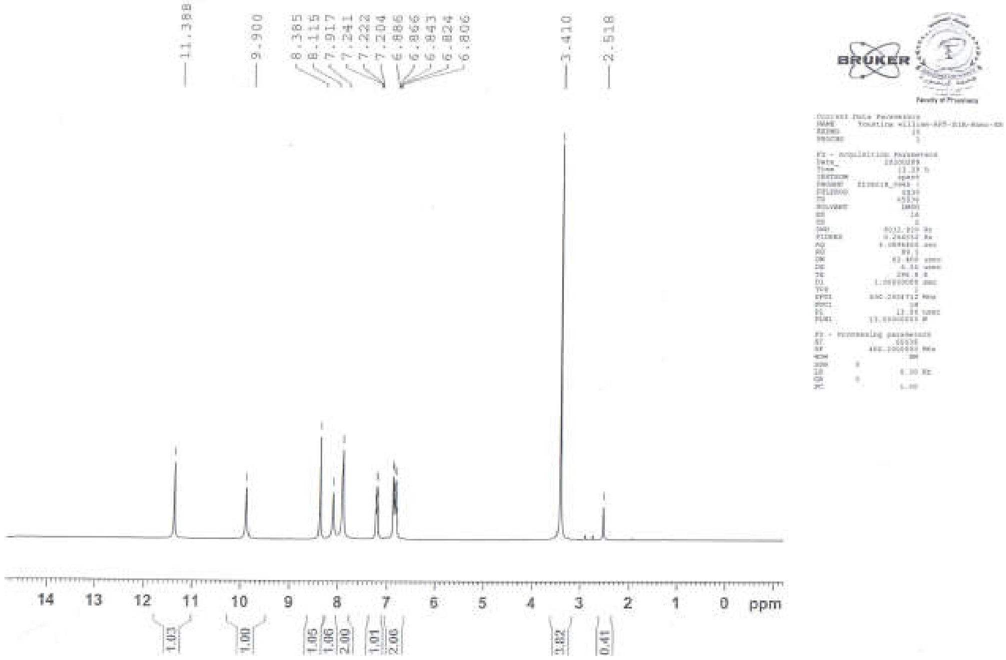

Fig. 3: ^{1}H-NMR spectrum of compound 3b

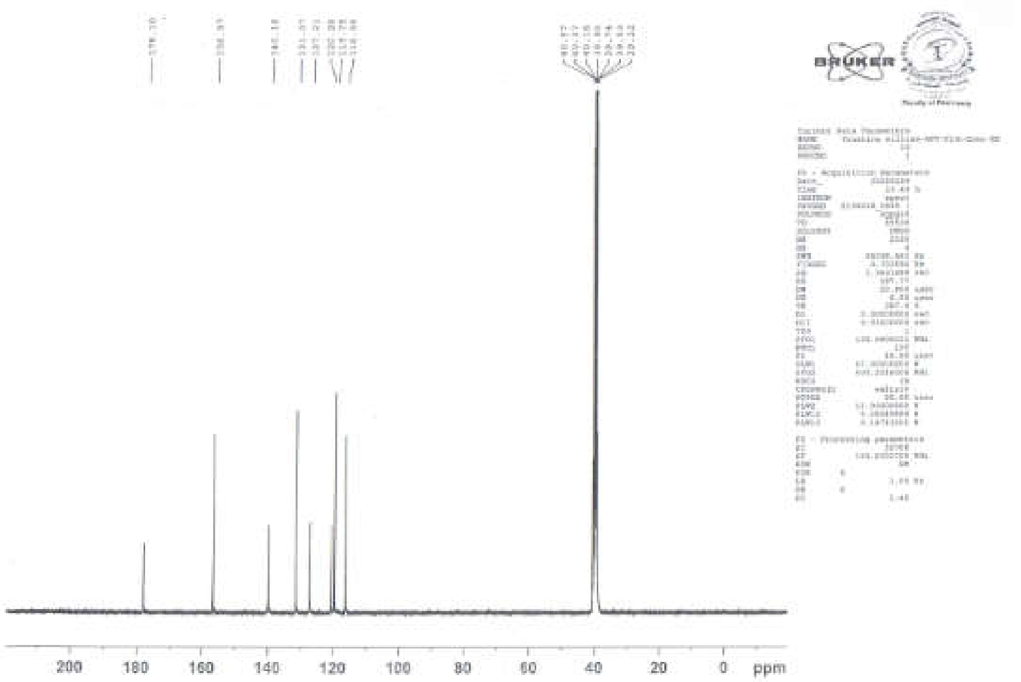

Fig. 4: ^{13}C-NMR spectrum of compound 3b

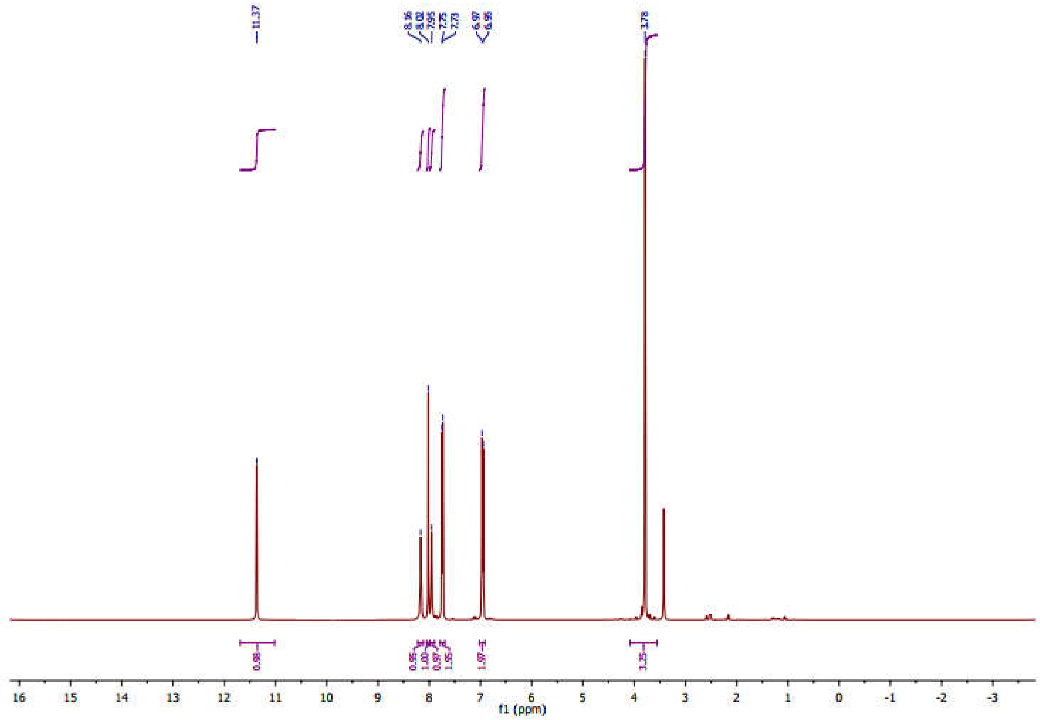

Fig. 5: ^{1}H-NMR spectrum of compound 3c

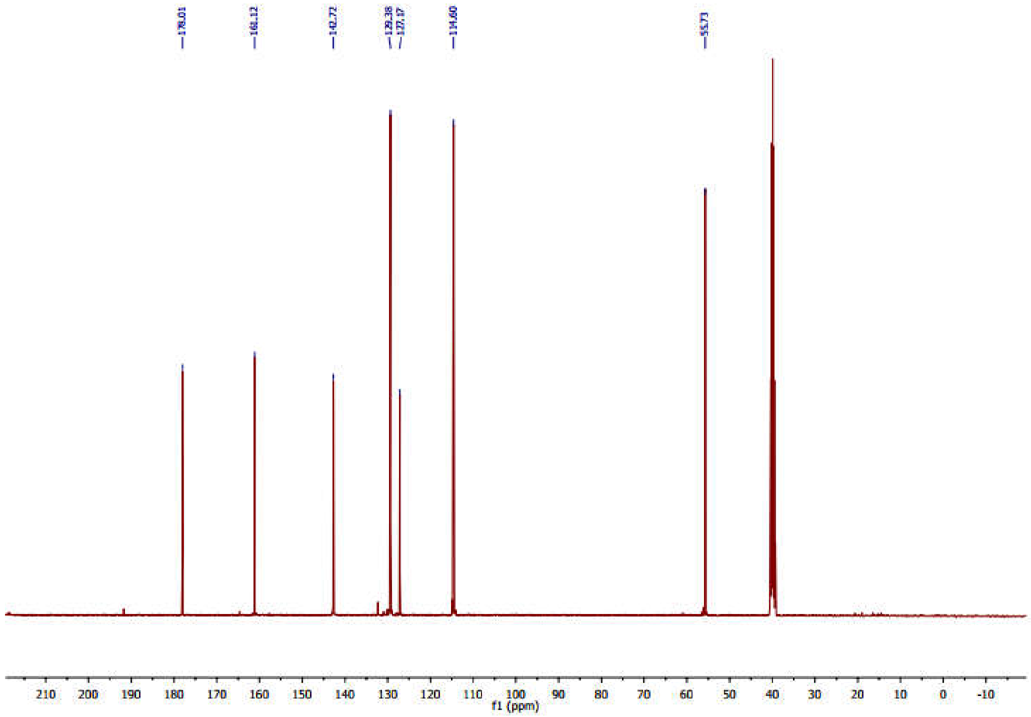

Fig. 6: ^{13}C-NMR spectrum of compound 3c

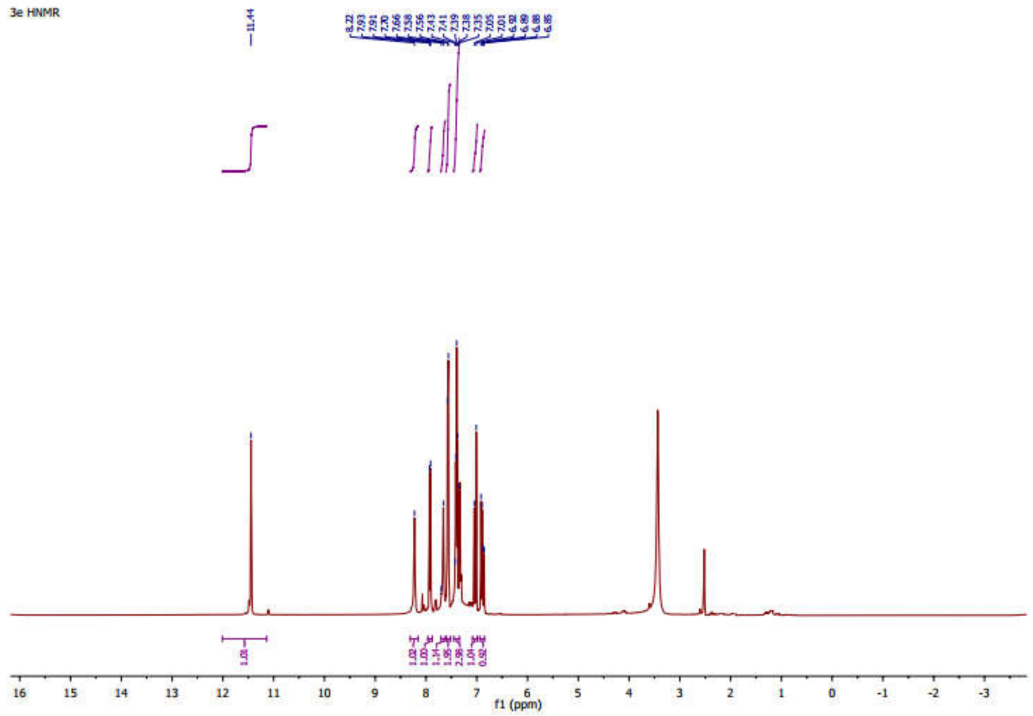

Fig. 7: ^{1}H-NMR spectrum of compound 3d

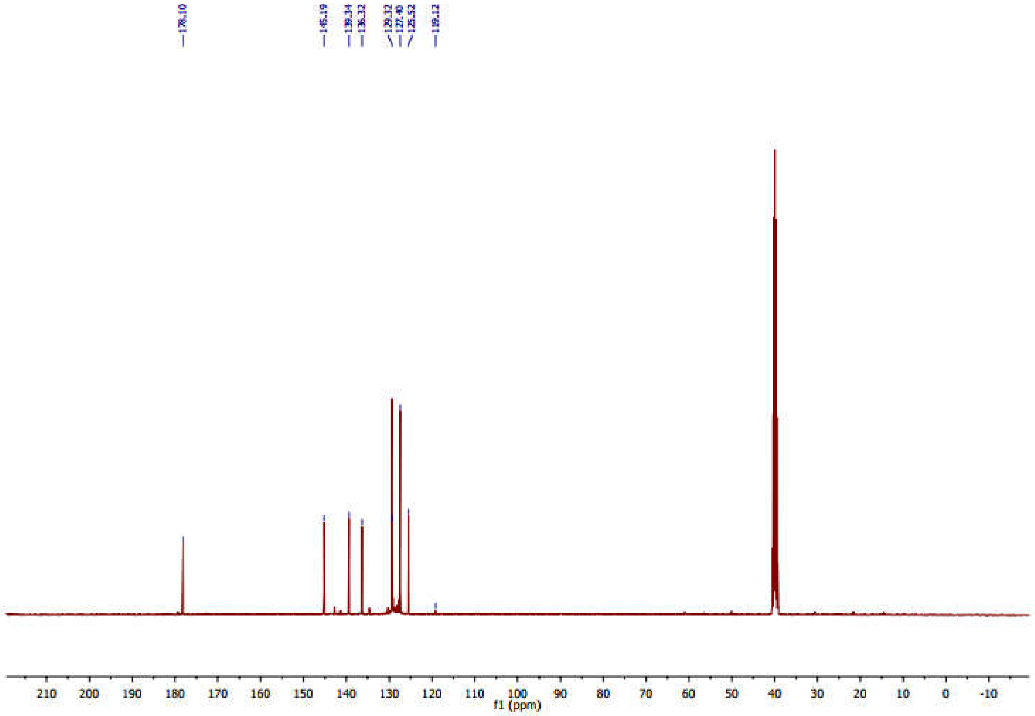

Fig. 8: ^{13}C-NMR spectrum of compound 3d

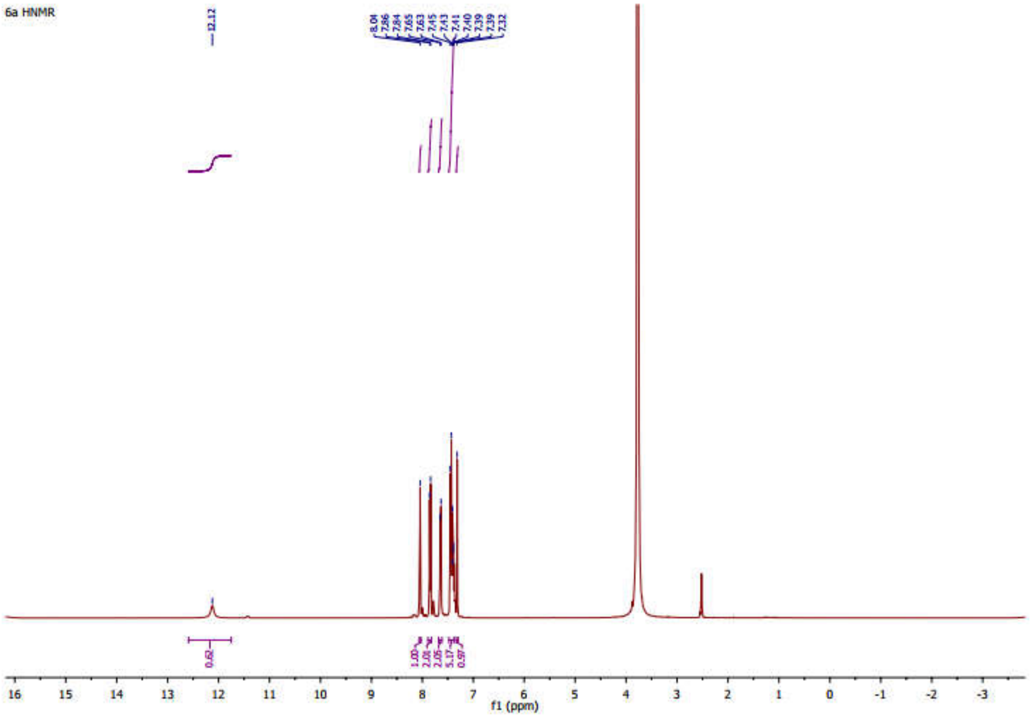

Fig. 9: ^{1}H-NMR spectrum of compound 4a

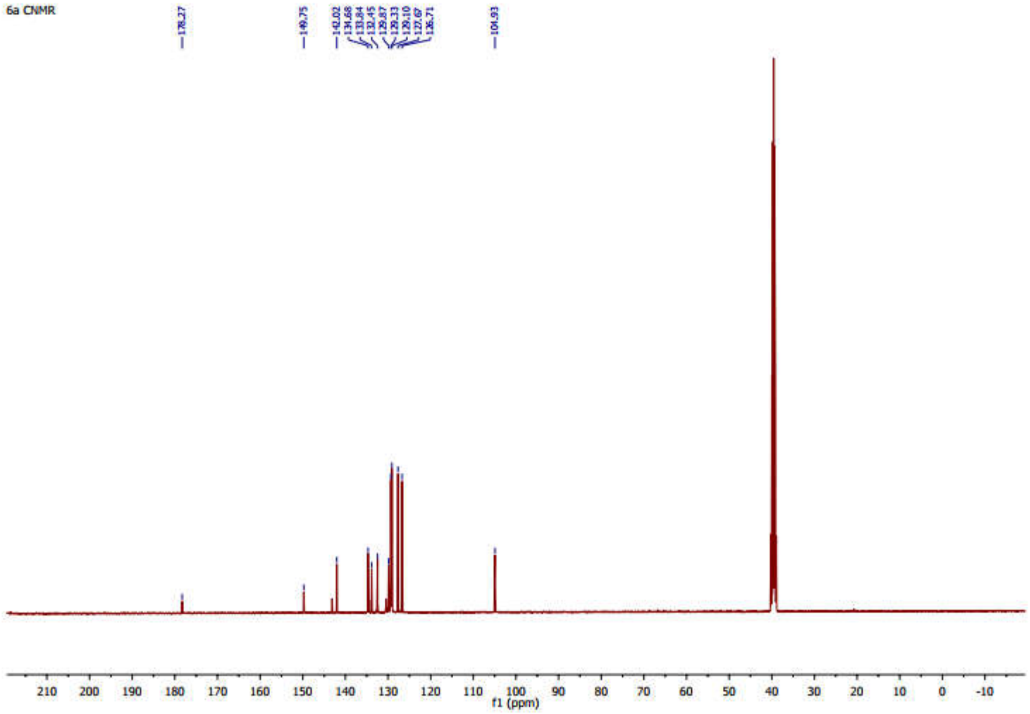

Fig. 10: ^{13}C-NMR spectrum of compound 4a

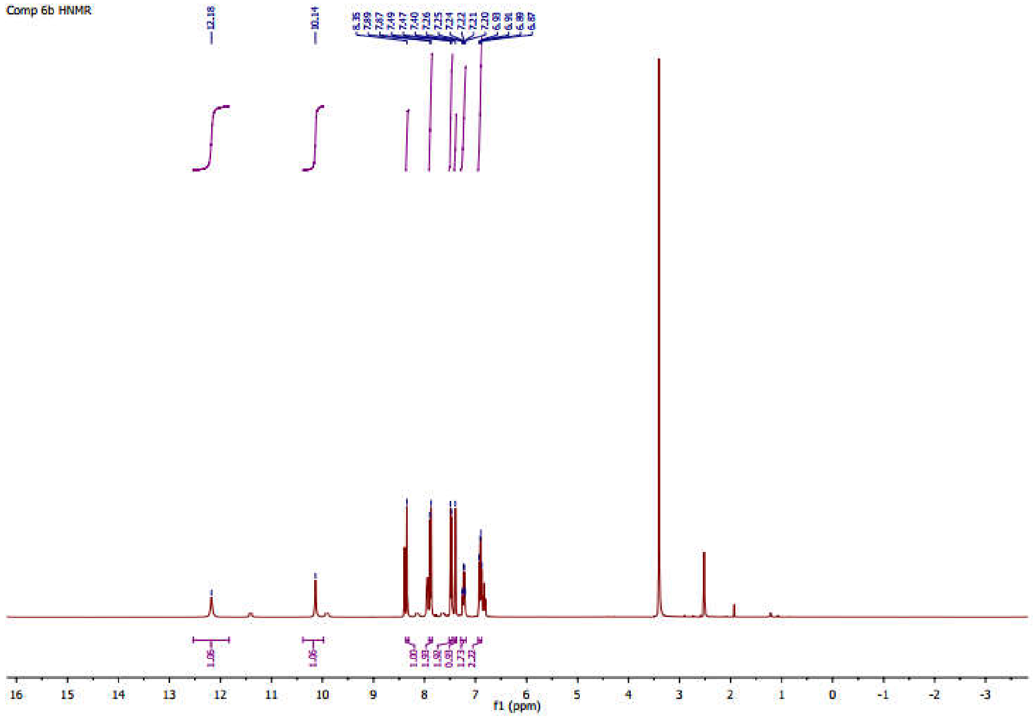

Fig. 11: ^{1}H-NMR spectrum of compound 4b

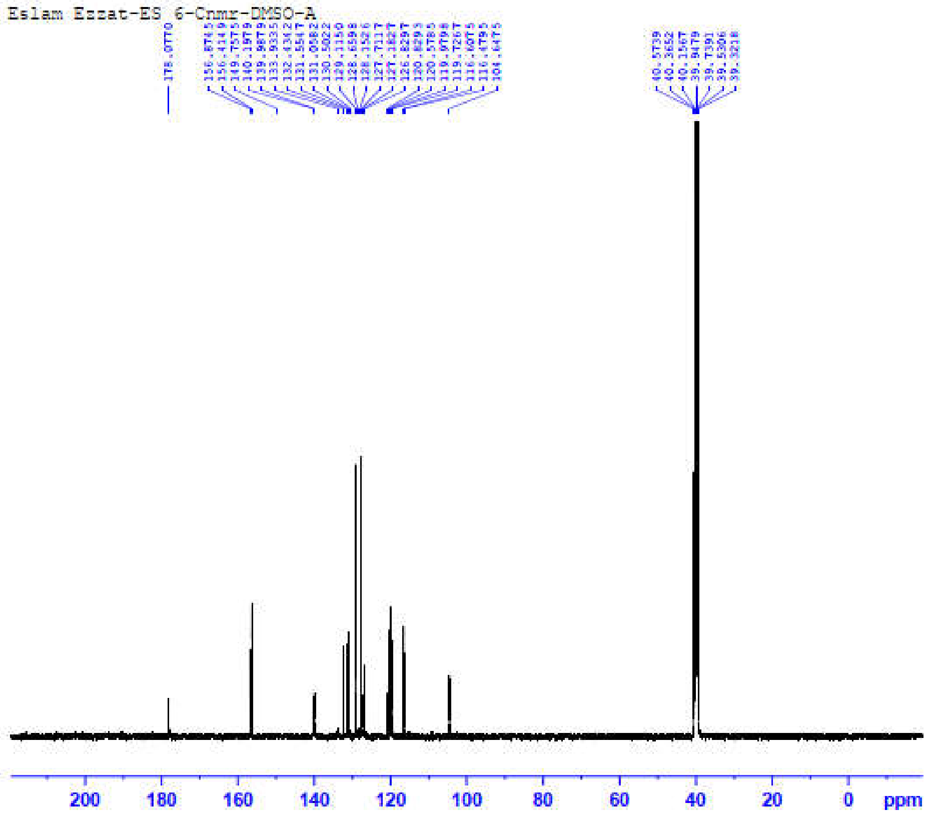

Fig. 12: ^{13}C-NMR spectrum of compound 4b

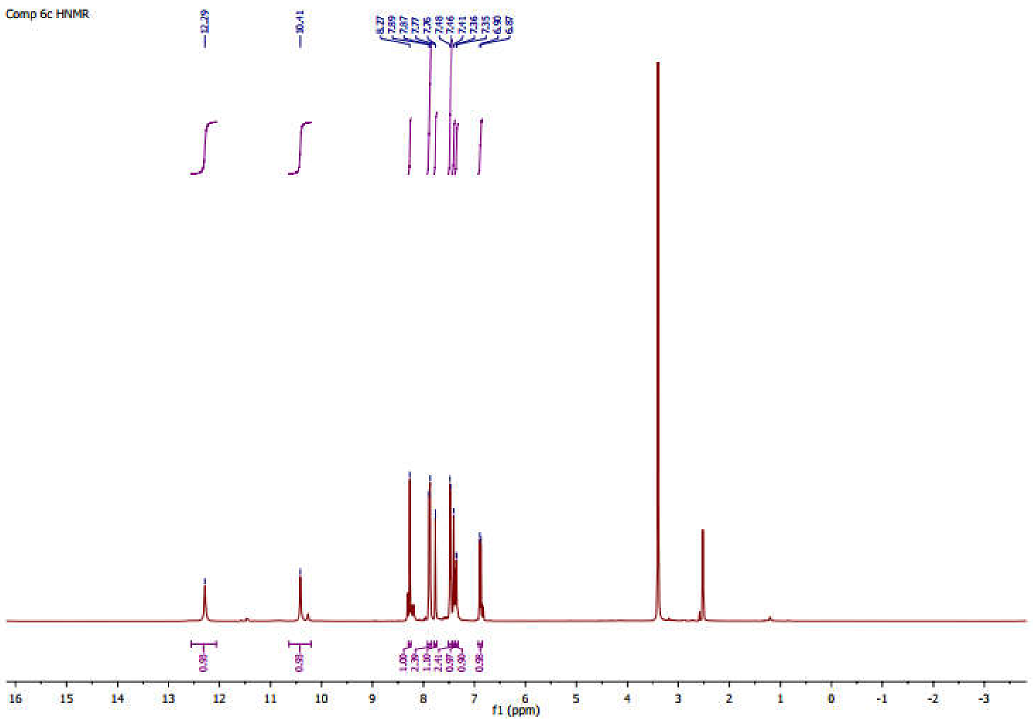

Fig. 13: ^{1}H-NMR spectrum of compound 4c

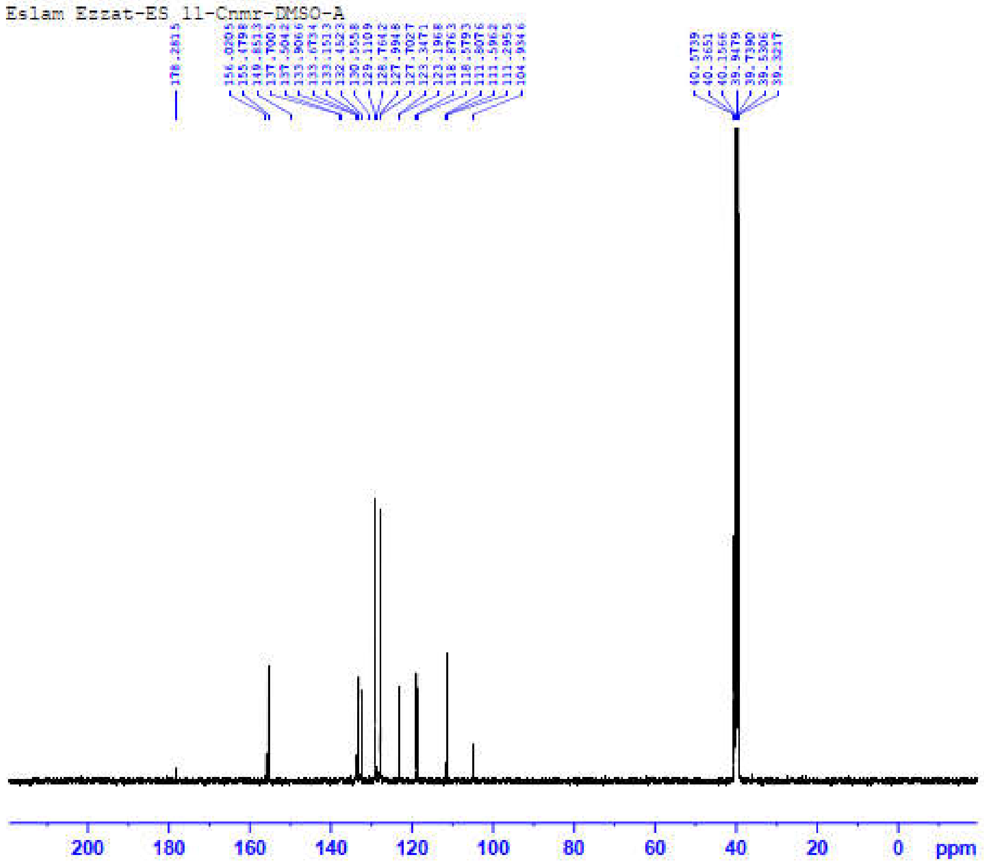

Fig. 14: ^{13}C-NMR spectrum of compound 4c

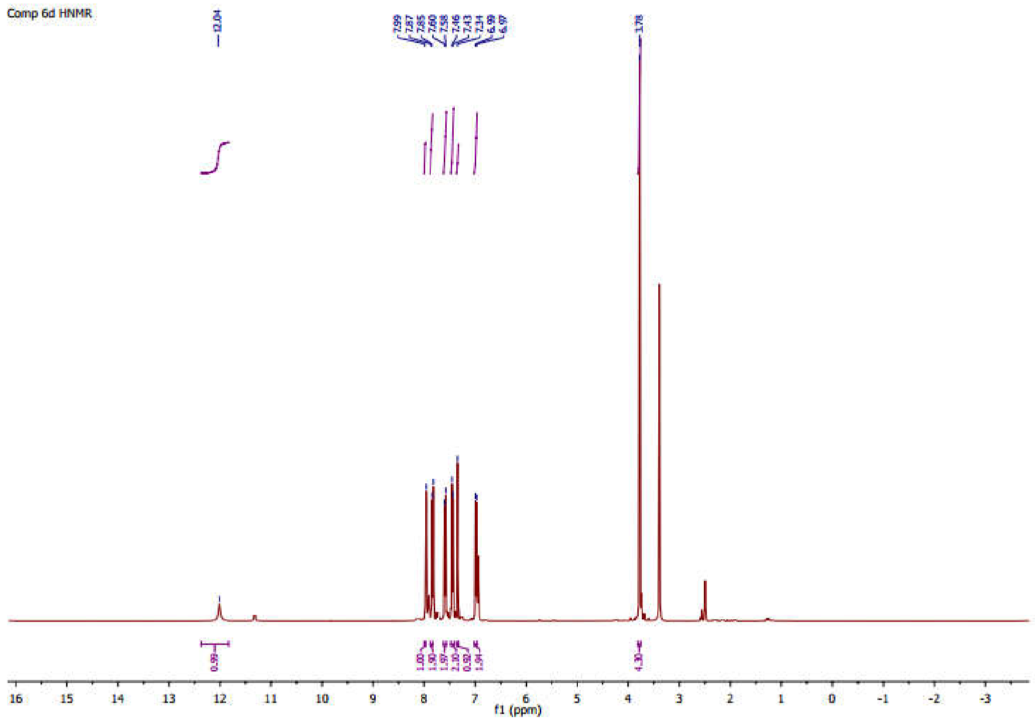

Fig. 15: ^{1}H-NMR spectrum of compound 4d

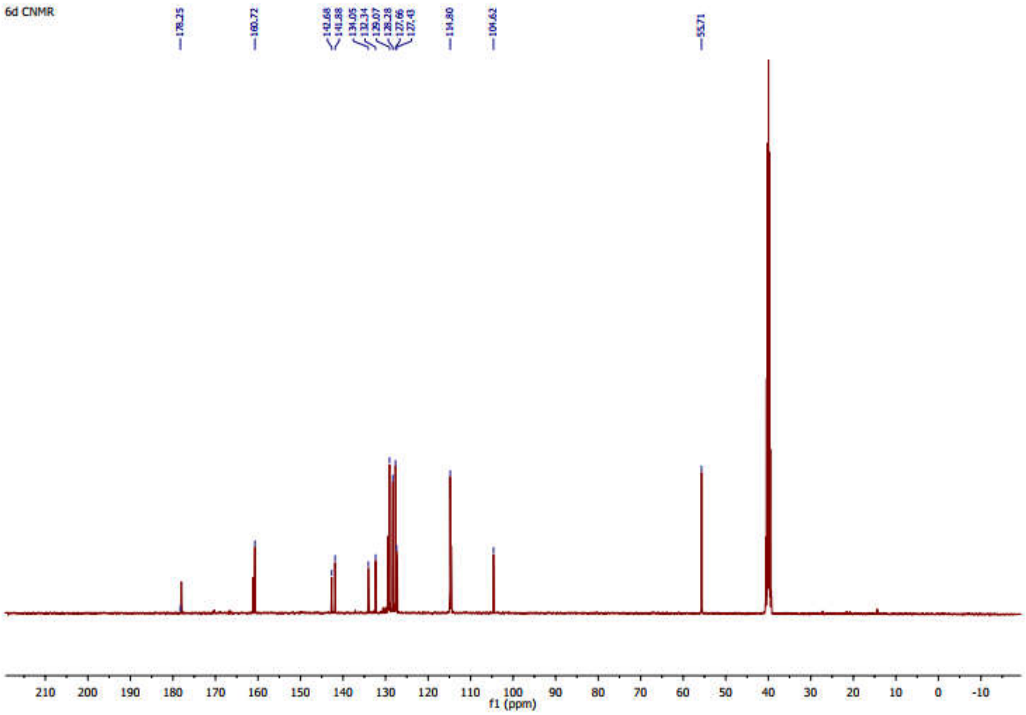

Fig. 16: ^{13}C-NMR spectrum of compound 4d

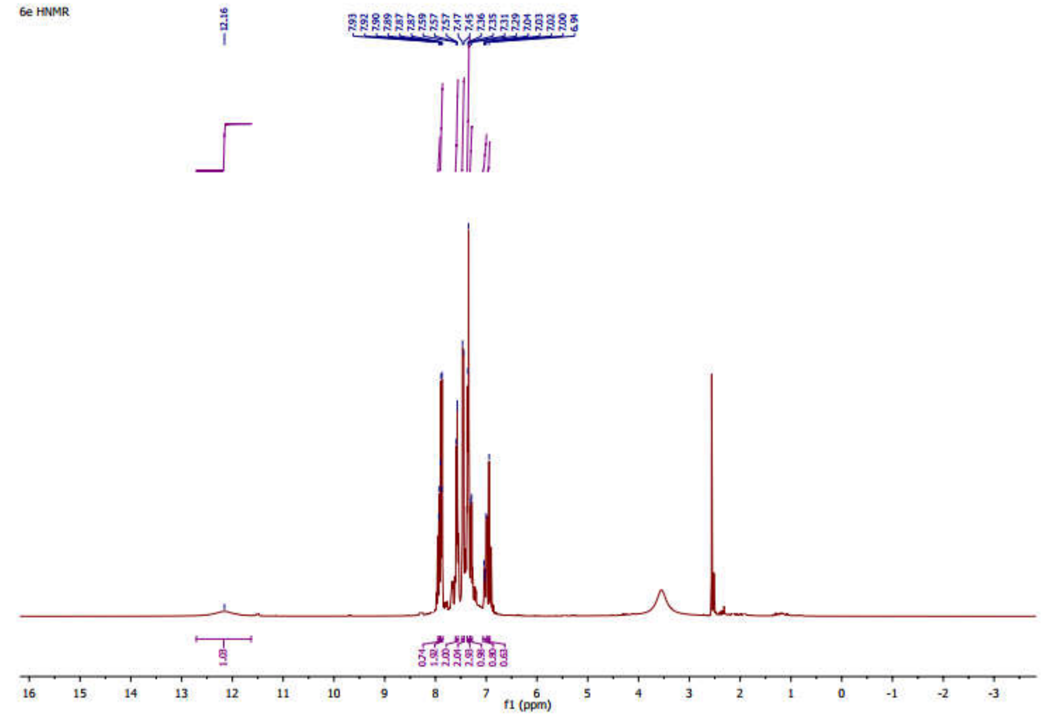

Fig. 17: ^{1}H-NMR spectrum of compound 4e

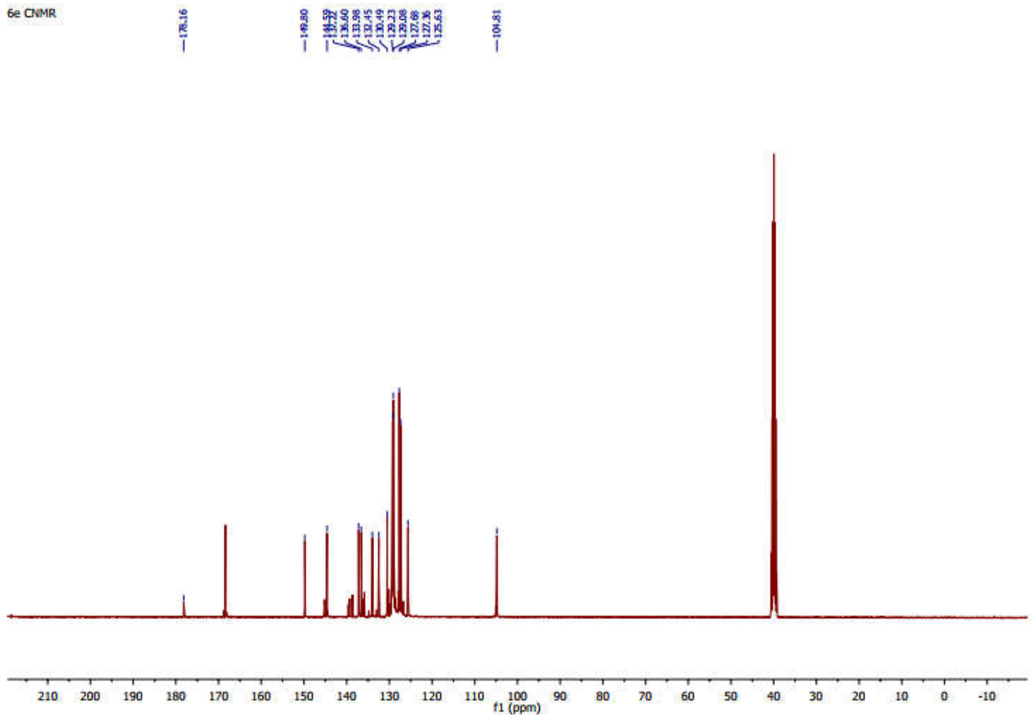

Fig. 18: ^{13}C-NMR spectrum of compound 4e

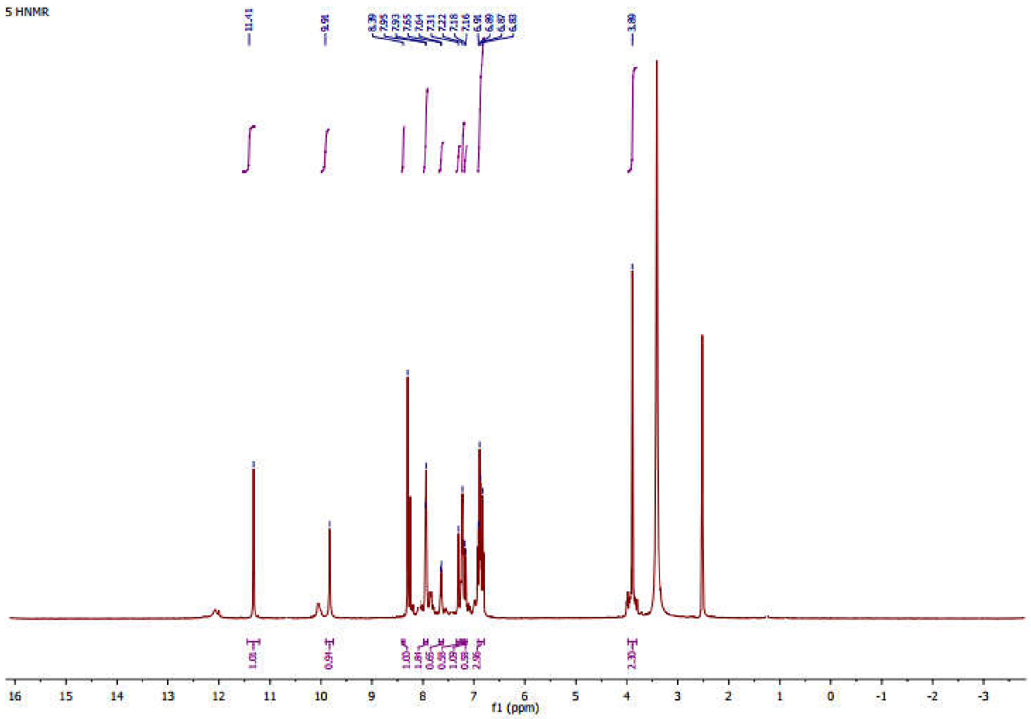

Fig. 19: ^{1}H-NMR spectrum of compound 5

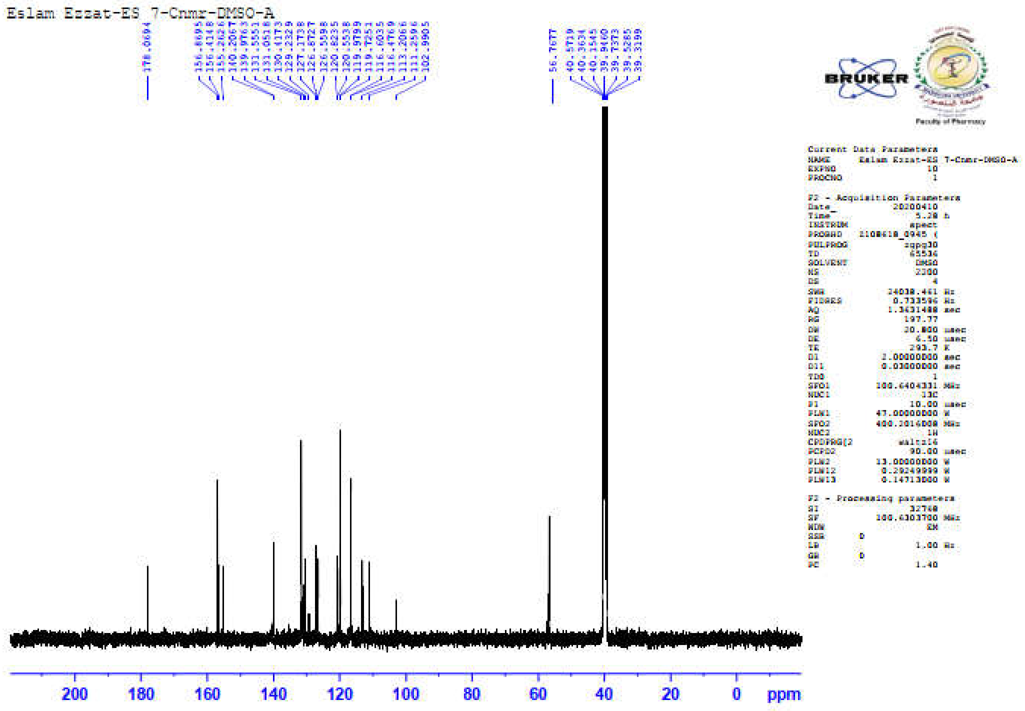

Fig. 20: ^{13}C-NMR spectrum of compound 5

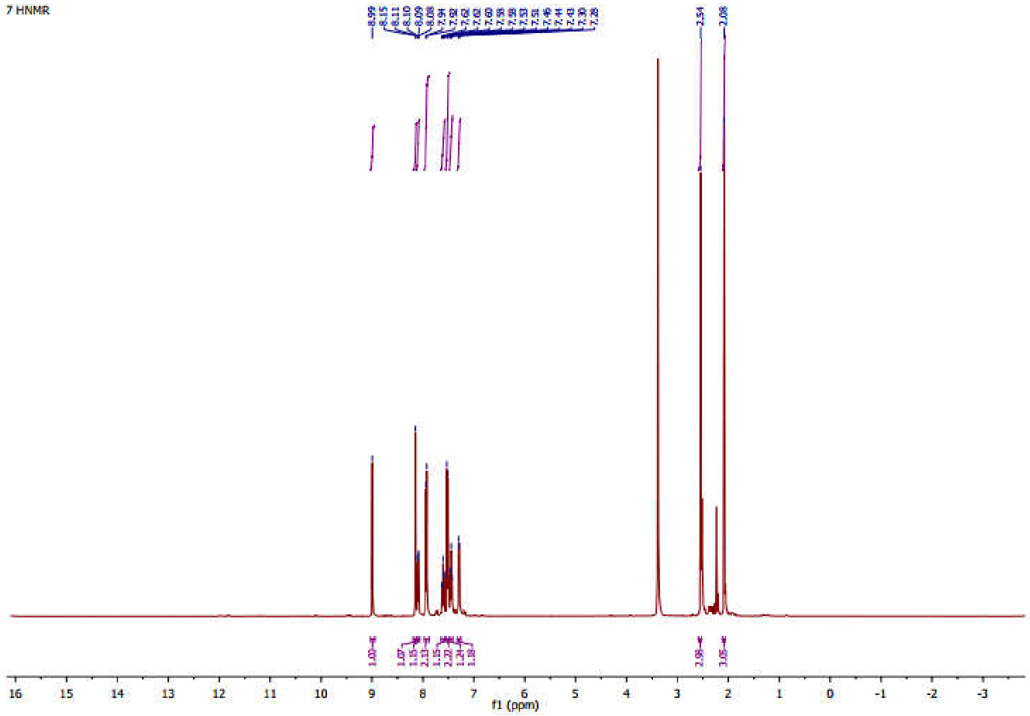

Fig. 21: ¹H-NMR spectrum of compound 6

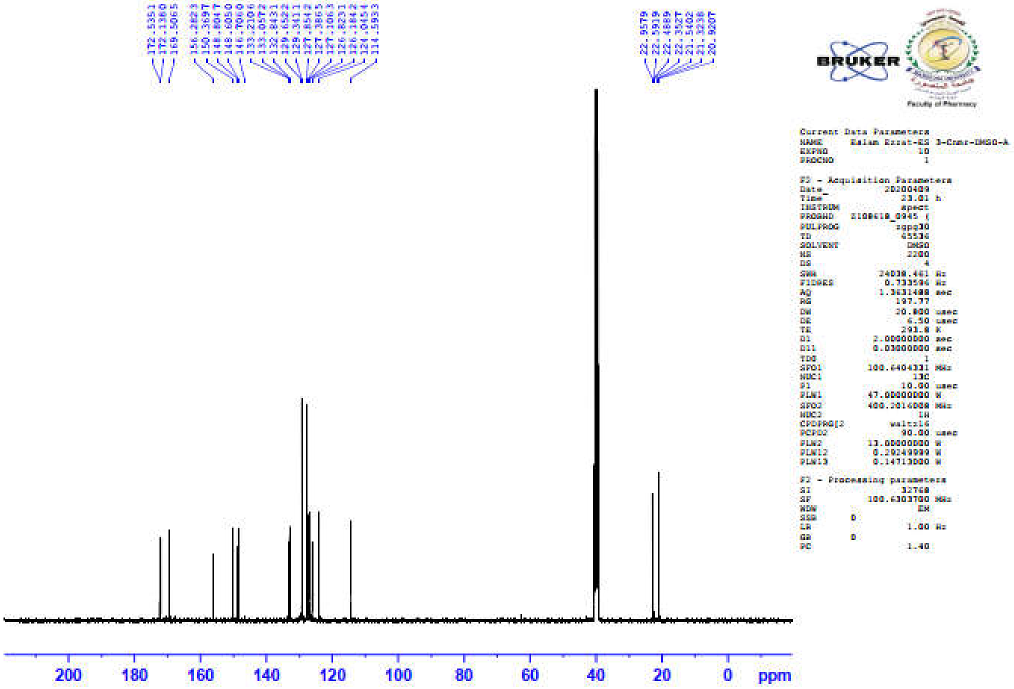

Fig. 22: ^{13}C-NMR spectrum of compound 6
